# The effect of cannabinoid type Ⅱ receptor on the excitability of substantia nigra dopaminergic neurons

**DOI:** 10.3389/fphar.2025.1522210

**Published:** 2025-02-14

**Authors:** Sha Zhao, Shunfeng Liu, Yongxin Gong, Zegang Ma

**Affiliations:** ^1^ Department of Physiology, School of Basic Medicine, Qingdao University, Qingdao, China; ^2^ Institute of Brain Science and Disorders, Qingdao University, Qingdao, China

**Keywords:** cannabinoid type II receptor, JWH133, AM630, substantial nigra, dopamine neurons, firing activity

## Abstract

The biological effects of cannabinoids are mainly mediated by two members of the G-protein-coupled-receptor family: cannabinoid type 1 receptor (CB1R) and cannabinoid type 2 receptor (CB2R). Unlike CB1R, CB2R is considered a “peripheral” cannabinoid receptor. However, recent studies have found that CB2R is widely expressed in the central nervous system and is involved in dopamine related behavioral regulation, including dietary behavior, weight regulation, anxiety, and schizophrenia like behavior. Our previous laboratory research demonstrated that activating CB2R on dopaminergic neurons in the ventral tegmental area can regulate addictive behavior in animals by inhibiting neuronal excitability. However, it is currently unclear whether CB2R on dopaminergic neurons in the substantia nigra compacta (SNc) has similar therapeutic potential. Brain patch clamp results have shown that the CB2R agonist JWH133 significantly inhibits the discharge of SNc dopamine neurons in a concentration dependent manner. The pharmacological blocker AM630 of CB2R can reverse this inhibitory effect, indicating that the expression of CB2R in SNc dopaminergic neurons is functional. After treatment with JWH133, the number of induced action potentials decreased, and the peak potential interval time, action potential start time, and potential amplitude after hyperpolarization amplitude all increased. In addition, synaptic current results showed that JWH133 can significantly reduce the frequency of miniature excitatory postsynaptic currents, indicating that activating CB2R to some extent inhibits the release of presynaptic glutamate and indirectly excites postsynaptic neurons.

## 1 Introduction

Hemp is a fiber flowering plant containing more than 500 components. One hundred four cannabinoids have been identified (Lafaye et al., 2017; Guo et al., 2024). The psychoactivity of cannabis is mainly mediated by cannabinoids. Cannabinoid 1 receptor (CB1R) and cannabinoid 2 receptor (CB2R) are the two main subtypes of cannabinoid receptors (Di Marzo, 2009; Brown et al., 2024). CB1R is a G-protein-coupled-receptor (GPCR) in the mammalian brain. The application of *CNR*1 mutant mice to study the effect of CB1R has significantly improved understanding of the function and mechanism of CB1R (Monory et al., 2015; Fyke et al., 2021; Yang et al., 2021). CB1R is mainly expressed in the central nervous system and hence is called central cannabinoid receptor (Cristino et al., 2020; Ruiz-Contreras et al., 2022). In 1993, researchers cloned CB2R and confirmed that CB2R is also a type of GPCR (Munro et al., 1993). Whether CB2R is expressed in the central nervous system and its function are not yet fully understood (Duerr et al., 2019; Jordan and Xi, 2019). Compared with CB1R, many characteristics of CB2R, such as structure, regulation, function, variation, and effect, on behavior need to be further studied (Xie et al., 2004; Grabon et al., 2023; Li et al., 2023). Previous studies have shown that CB2R is also expressed in the brain, although its expression level is far lower than that of CB1R in healthy subjects. Immunohistochemistry and *in situ* hybridization were used to detect the expression of CB2R immunoreactive cells or CB2R mRNA in various brain regions (Galiègue et al., 1995). CB2R was detected in glutamate neurons in the hippocampus (Zhao et al., 2010; Komorowska-Müller et al., 2021), pyramidal neurons in the cortex (den Boon et al., 2014; Uzuneser et al., 2023), dopaminergic neurons in ventral tegmental area (VTA) (Liu et al., 2017; Zhang et al., 2017; Zhang H. et al., 2021), neurons in the nucleus accumbens (NAc) (Li et al., 2021; Feng et al., 2023), and the brainstem and small brain (Ashton et al., 2006; Baek et al., 2008). Therefore, attention has been paid to the function and mechanism of CB2R in neurons.

Basal ganglia is one of the brain regions with high expression of CB2R (Sagredo et al., 2007; Soti et al., 2022). CB2R is expressed in the globus pallidus (Lanciego et al., 2011), VTA, and subthalamic nucleus (Sánchez-Zavaleta et al., 2018; Ma et al., 2019). The relative expression of CB2R in midbrain dopaminergic neurons is high, which can help regulate a variety of dopamine (DA)-related behaviors (Canseco-Alba et al., 2021). CB2R regulates food intake, weight, depression, anxiety, and schizophrenia like behavior, and also plays an important role in cocaine, alcohol, and nicotine addiction (Onaivi et al., 2012; Huang et al., 2016; Delis et al., 2017; Araujo et al., 2019). Activation of CB2R on VTA dopaminergic neurons inhibits the cAMP-protein kinase A (PKA) signal transduction pathway in neurons, enhance muscarinic type K^+^ current (M-current), and inhibit the excitability of neurons (Ma et al., 2019). *In vivo* voltammetry was used to record endogenous DA release from dopaminergic terminals. CB2R activation inhibited presynaptic dopamine release and induced antipsychotic effect of muscarinic M4 acetylcholine receptor positive allosteric modulator (Zhang et al., 2017; Li et al., 2021). In addition, overexpression of CB2R in the brain inhibits cocaine self-administration and regulates cocaine induced motor behavior (Aracil-Fernández et al., 2012). Thus, CB2R plays an indispensable role in the regulation of dopamine related animal behavior and various brain functions (including psychiatric, cognitive, and neurobiological activities) related to dopamine.

At present, the study of CB2R on substantia nigra compacta (SNc) dopaminergic neurons is very limited. The changes of excitability of SNc dopaminergic neurons can affect DA content in striatum and thus help regulate the SNc striatum system (Gonon, 1988; Gantz et al., 2018). CB2R is expressed in both soma and axon terminals of dopaminergic neurons in SN of rats (Shi et al., 2017). Moreover, CB2R on axon terminals can increase DA release by interacting with the presynaptic D2 receptor (Amancio-Belmont et al., 2020; Zhang H. Y. et al., 2021). Therefore, exploring the effect of activation of CB2R on the electrical activity of SNc dopaminergic neurons, as well as its regulatory mechanism, is essential to further understand the function of CB2R on dopaminergic neurons. The aim of this study was to investigate the effect of activation of CB2R on SNc dopaminergic neurons on neuronal excitability and its possible mechanism.

CB2R expression in SNc dopaminergic neurons of mice was first observed using immunofluorescence technique and the whole cell patch clamp technique was used to observe the effect of activation of CB2R on the excitability of SNc dopaminergic neurons. The effect of CB2R on the excitability of SNc dopaminergic neurons and its possible mechanism were evaluated in this study from the molecular and electrophysiological perspectives, and its possible physiological significance was elucidated.

## 2 Materials and methods

### 2.1 Animals

C57BL/6j mice were purchased from Beijing Vital River Laboratory Animal Technology Co., Ltd. The mice were placed in the laboratory for 2 h for adaptation and then housed in separate cages. The mice were placed in an environment with a constant humidity and temperature of 50% ± 10% and 21°C ± 2°C, respectively. The mice were also subjected to a day: night cycle of 12:12 h and ad/lib feed and water for the duration of the study. The C57/Bl6j male mice aged 15–20 days were used for the preparation of the SNc brain slices. Additionally, C57BL/6j male mice aged 7–8 weeks were used for double immunofluorescence staining. The animal feeding and experimental procedures in this experiment were conducted strictly in accordance with the Qingdao University Laboratory Animal Use Regulations.

### 2.2 Double immunofluorescence staining

To avoid estrogen interference, four male mice were selected for immunofluorescence experiments (Kim et al., 2022; Kim et al., 2023). Male mice were anesthetized with pentobarbital sodium (70 mg/kg, i. p., dissolved in saline) and administered physiological saline and 4% paraformaldehyde solution through the heart. The brains were removed and fixed in a 4% paraformaldehyde solution, and then dehydrated in 20% and 30% sucrose solutions at 4°C sequentially. Subsequently, the brains were frozen and sectioned into 30 μm slices for immunofluorescence staining. The slices were incubated with 5% bovine serum albumin (BSA) for 1 h to block non-specific binding, before incubation with primary antibodies containing mouse anti-tyrosine hydroxylase antibodies (CST, 1:1,000) and rabbit anti–CB2R antibodies (Abcam, 1:250) at 4°C for 12 h. Secondary antibodies (goat anti-mouse immunoglobulin G (IgG) (Abcam, 1:500); goat anti-rabbit IgG (Abcam, 1:500)) were then applied at room temperature for 1 h. Following incubation, the sections were washed, dehydrated, mounted on polylysine-coated glass slides, and examined using a fluorescence microscope (BX 53, Olympus, Tokyo, Japan). Sections were also observed using an Olympus fluorescence microscope (VS120, Olympus, Tokyo, Japan) and 20x or 40x objective lens micrographs obtained.

### 2.3 SNc slice preparation

In the present study, postnatal day 15 (P15) to P20 male mice were used as no significant changes in CNR2 mRNA levels are observed in male mice during P15–P40 (Dufour et al., 2014). The slice patch-clamp recordings were the same as reported previously (Chang et al., 2020). Briefly, the mice were anesthetized before decapitation, and slices were cut in an ice-cold, oxygenated cutting solution containing (in mM) 124 NaCl, 3 KCl, 0.5 CaCl_2_, 2 MgCl_2_, 1.3 NaH_2_PO_4_, 26 NaHCO_3_, and 10 glucose. Coronal slices (250 μm) containing SNc were cut using a vibratome (LeicaVT1000S, Germany) and transferred to a holding chamber. The slices were the incubated at 36 °C in artificial cerebrospinal fluid (ACSF) containing (in mM) 124 NaCl, 3 KCl, 1.3 NaH_2_PO_4_, 1.3 MgCl_2_, 2.4 CaCl_2_, 26 NaHCO_3_, and 10 Glucose (osmolarity, 300–310 mM, pH 7.4), and continuously bubbled with 95% O_2_ and 5% CO_2_. After 1 h of recovery, the slices obtained were then transferred to an imaging chamber on the stage of an upright microscope (BX51WI; Olympus Tokyo, Japan) and continuously perfused with oxygenated standard ACSF at a flow rate of 2 mL/min at RT.

### 2.4 Patch-clamp recordings in SNc slices

Whole-cell patch-clamp recordings were conducted using glass pipettes with a resistance of 3–7 MΩ. The internal solution (295 mOsm) contained (in mM): 120 K-gluconate, 10 HEPES, 10 EGTA, 20 KCl, 2 MgCl_2_, 2 Na_2_ATP, 0.3 Na_3_GTP, and 10 Na_2_-phosphocreatine; pH 7.2 with KOH. Cells were visualized under infrared differential interference contrast (DIC) microscopy, and electrodes were positioned using a micromanipulator. After a tight seal (resulting in electrode resistance >1 GΩ) was formed between the electrode tip and the cell surface, suction was briefly applied until a whole-cell patch-clamp recording configuration was obtained (access resistance lower than 20 MΩ). Recordings started at least 5 min after establishing whole-cell configuration to allow the proper wash-in of the intra-pipette solution. Whole-cell patch clamp recordings of SNc neurons was conducted to record spikes under current-clamp mode. Step-current injections (of 800 milliseconds) were delivered from 0 pA to 50 pA increments. The number of action potentials (AP), AP initiation, AP duration, after-hyperpolarization (AHP), and inter-spike interval (ISI) between the first and second firings were measured in current mode with the cell at −70 mV and were analyzed with a custom Matlab program (R2018b).

### 2.5 Miniature excitatory/inhibitory postsynaptic currents (mEPSCs/mIPSCs) in SNc slices

For assessments of mEPSCs, electrodes were filled with an intracellular solution containing (mM): 133 K-gluconate, 8 NaCl, 0.6 EGTA, 2 MgATP, 0.3 Na_3_·GTP, and 10 HEPES. To allow verification of the identity of recorded neurons, sodium channel blocker tetrodotoxin (TTX 1 μM), D-APV (a NMDA receptor antagonist, 50 μM) and picrotoxin (a selective GABA_A_ receptor antagonist) 100 μM were included in the solution. The membrane potential was clamped at −70 mV and the mEPSCs was recorded for 15 min. For mIPSCs recordings, electrodes were filled with an intracellular solution containing (mM): 130 KCl, 1.0 MgCl_2_, 5 EGTA, 5 Na_2_·ATP, and 5 HEPES. To allow for verification of the identity of recorded neurons, TTX, NBQX (an AMPA receptor antagonist, 10 μM) and D-APV (50 μM) were included in the solution. The membrane potential was clamped at −60 mV, recording time were the same as above.

### 2.6 Electrophysiology data acquisition and analysis

Total 53 mice were used in electrophysiological experiments, we maintained two slices from each mice and then selected one neuron for patch recordings based on the brain slice. Only the neurons with the morphology and electrophysiological properties of dopaminergic neurons in SNc were included for further analysis. Series resistance was automatically compensated using a patch-clamp amplifier (Multiclamp 700 B, Molecular Devices). Data acquisition and analysis were performed using a digitizer (DigiData 1550B, Molecular Devices) and a pClamp10 analysis software (Molecular Devices). Signals were filtered at 2 kHz and sampled at 10 kHz. Offline analyses of the data obtained from electrophysiological recordings were conducted using the Clampfit software version 10.7 (Axon Instruments, Inc., United States). A semi-automated sliding template protocol was utilized to analyze both classes of miniature data. The detection criteria were established by optimizing the scaling factor and ensuring an optimal fit quality. Events were confirmed when the criterion exceeded a predefined threshold level. Furthermore, the algorithm dynamically adjusted for variations in recording noise, considering only amplitudes that were at least three times the standard deviation of the noise (3σ). Each event detected by the template was individually evaluated and accepted for analysis based on two criteria: non-overlapping events and stable baseline recordings (2.5 ms), both before and after the rising and decay phases of mIPSCs/mEPSCs. Once validated, the mIPSCs/mEPSCs were aligned at their onset and averaged.

### 2.7 Drugs and statistics

JWH133 and AM630 were purchased from APE x Bio (United States). Other chemical drugs used for the electrophysiology experiments were obtained from Sigma. The Graphpad prism 5.0 software was used to statistically analyze the experimental results and to make the corresponding results analysis and trend graphs. The experimental data are expressed as mean ± standard error (Mean ± S.E.M.), and paired t-test of *P* < 0.05 was considered statistically significant.

## 3 Results

### 3.1 Nigral dopaminergic neurons expressed CB2Rs

**FIGURE 1:**
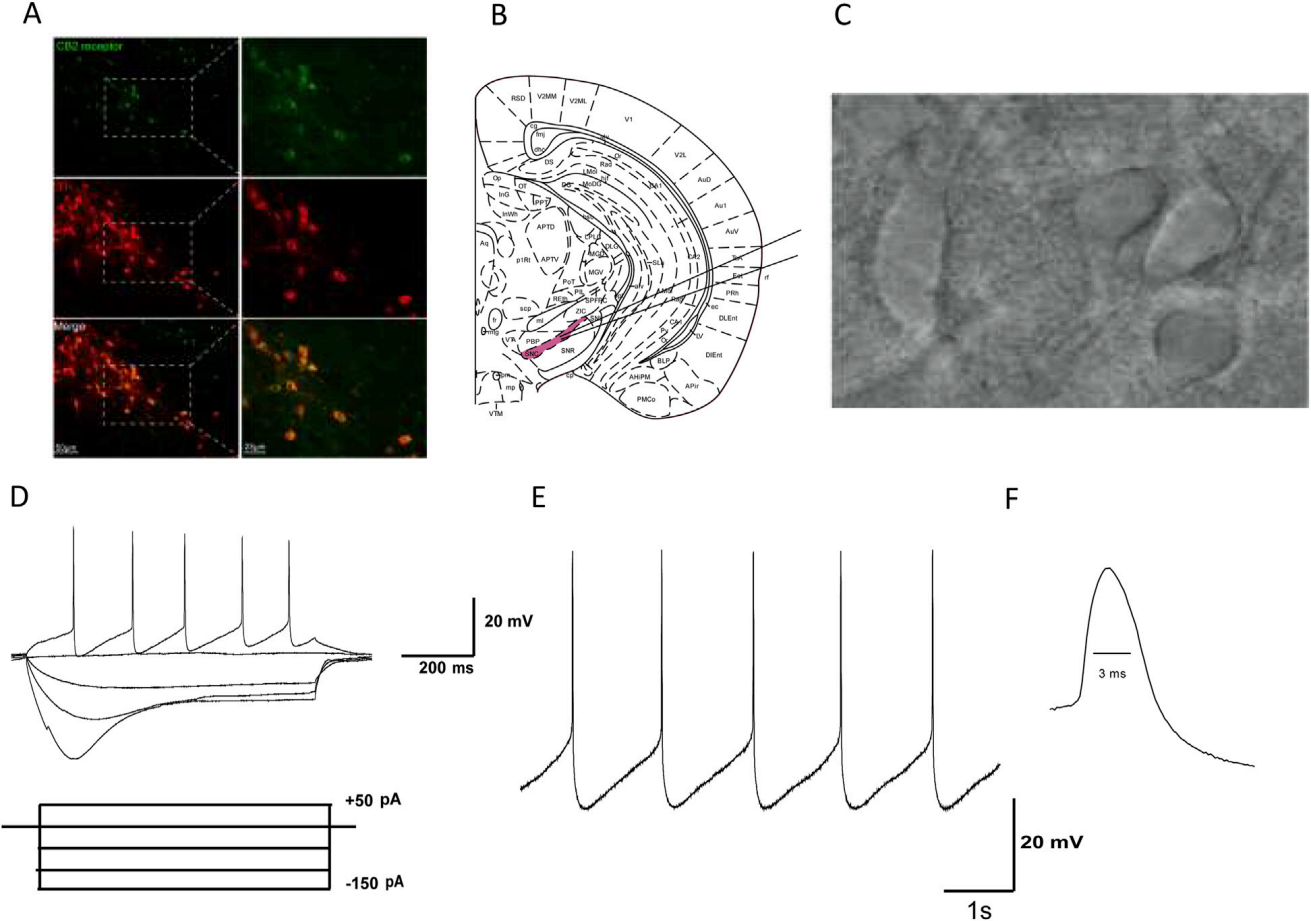
Identification of dopaminergic neurons in the SNc of mice. **(A)** Green fluorescence represents the expression of CB2R-positive cells (top line). Red fluorescence represents the expression of TH-immunostaining neurons (middle line). Pictures in the bottom line indicated the co-localization of CB2R and TH positive cells. **(B)** The substantia nigra is located at the ventral midbrain. **(C)** IR-DIC video microscope of SNc neurons in the midbrain slice. **(D)** Electrophysiological characteristics of substantia nigra dopaminergic neurons. Current clamp recordings showed spontaneous low-frequency pacemaker activity and inward rectification property by injection of −150 pA hyperpolarized current. **(E)** In Gap free mode, SNc dopaminergic neurons exhibit slow and regular spontaneous firing activity. **(F)** The duration of the action potential (AP) is relatively long, with a half width of about 3 ms.

Firstly, we observed the expression of CB2Rs on dopaminergic neurons in the SNc of normal mice. Double-labeling immunofluorescence showed that dopaminergic neurons in the substantia nigra expressed CB2Rs. As shown in Figure 1A, some dopamine neurons in the substantia nigra labeled with TH (red) also exhibit CB2R immunoreactivity (green).

### 3.2 Identification of dopaminergic neurons in mouse SNc brain slices

**FIGURE 2:**
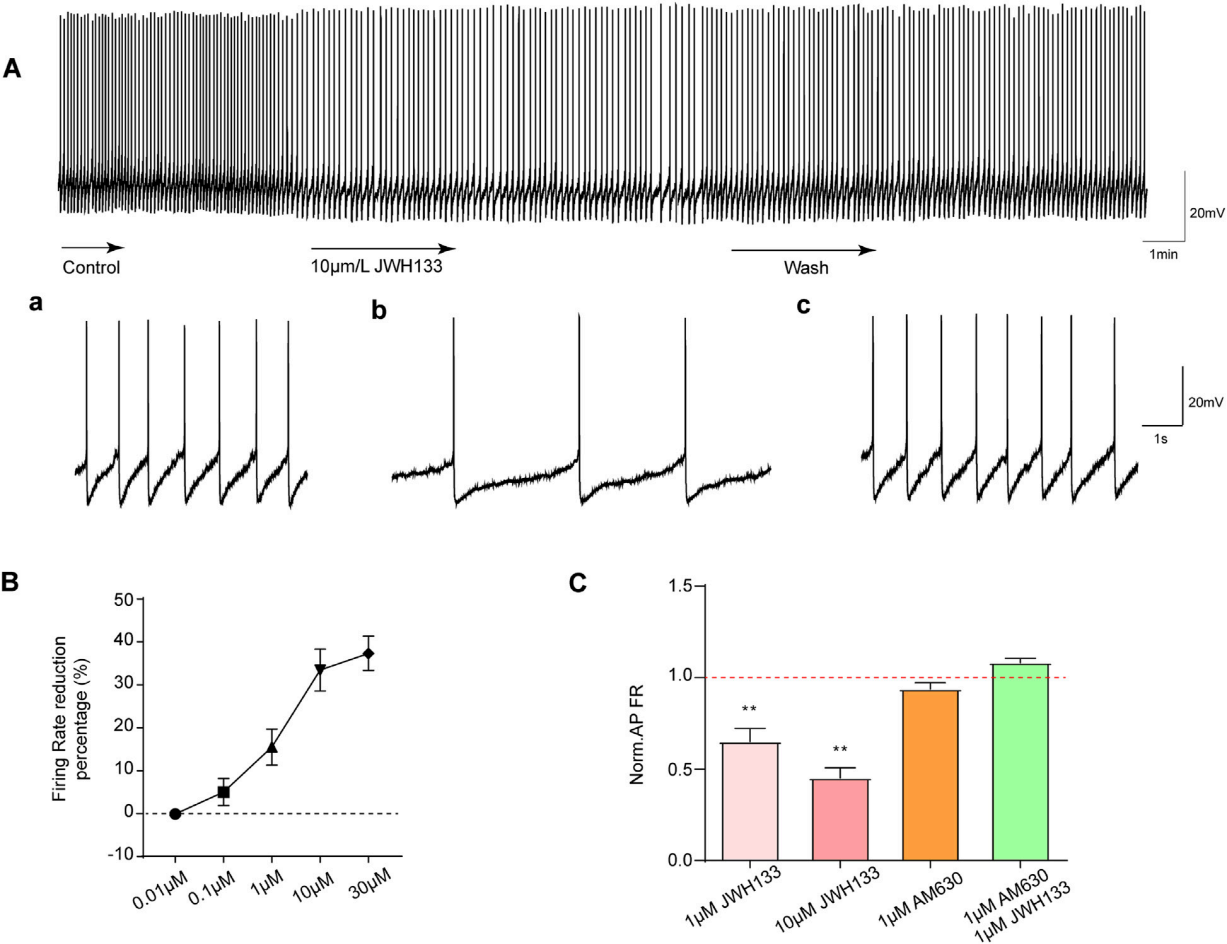
Effects of JWH133 on spontaneous firing of dopaminergic neurons in the SNc. The activation of CB2R reduces the discharge of dopamine neurons *in vitro*. **(A)** JWH133 significantly reduces the firing rate of dopaminergic neurons in the SNc. Representative pictures sowed the firing before (a), after JWH133 perfusion (b) and wash (c). **(B)** and **(C)** Summarized data showed that JWH133 dose-dependently inhibits firing frequency of dopamine neurons. The inhibitory effect of JWH133 on firing frequency was blocked by AM630 (1 μM) (n = 7, **P < 0.01, compared with control).

**FIGURE 3:**
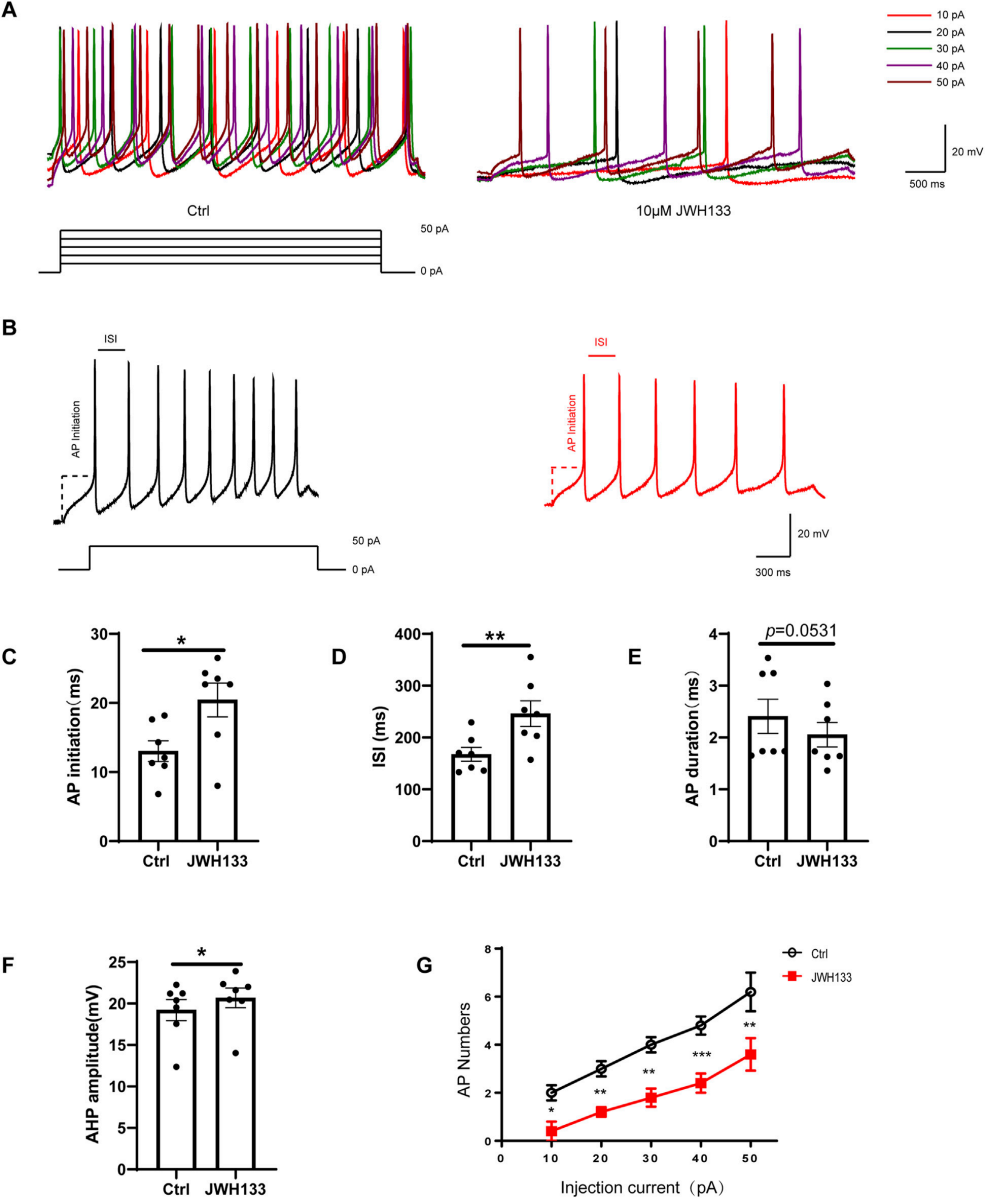
Effect of JWH133 on evoked firing of dopaminergic neurons in the SNc. Representative recordings **(A)**and summarized data **(B)** illustrated that JWH133 reduces evoked firing numbers of dopaminergic neuron when the neurons were injected 50 pA current. Representative recordings **(C)** and summarized data **(C–G)** illustrate that JWH133 increases ISI, AP initiation, AHP, and decreases AP numbers when neurons were injected with 50 pA current. (Figures 3C–F; paired t-test, ISI: t = 4.055, df = 6, *P* < 0.01; AP initiation: t = 3.392, df = 6, *P* < 0.05; AHP: t = 3.187, df = 6, *P* < 0.05; AP duration: t = 2.403, df = 6, *P* = 0.0531; AP number: t = 5.303, df = 6, *P* < 0.01). Data are presented as the mean ± SEM, compared with control.

**FIGURE 4:**
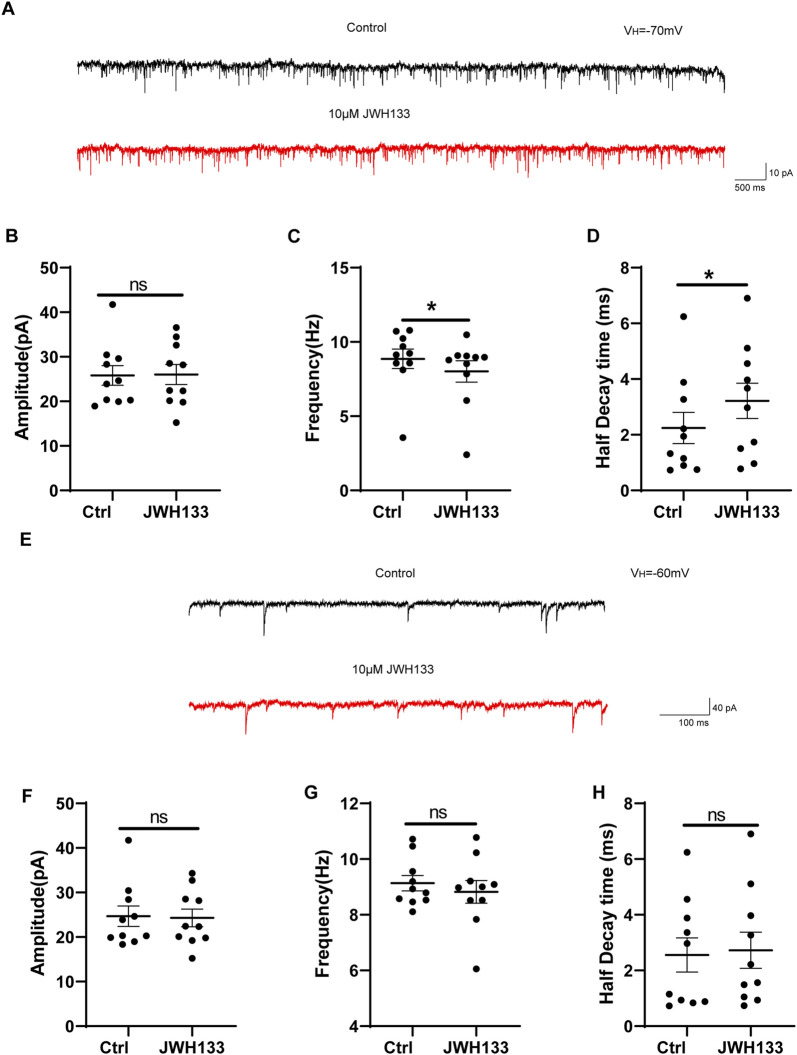
The regulation of JWH133 on miniature excitatory/inhibitory postsynaptic current. **(A)** Typical traces showed the mEPSCs in the presence of TTX (1 μm) before (top trace) and after (bottom trace) JWH133 exposure. **(B–D)** Statistical analysis showed the influence of JWH133 on the frequency (n = 10, t = 3.146, df = 9, *P* < 0.05, Figure 4C), half-decay time (n = 10, t = 2.370, df = 9, *P* < 0.05, Figure 4D) and amplitude (n = 10, t = 0.09425, df = 9, *P* > 0.05, Figure 4B) of mEPSCs. **(E)** Typical traces of mIPSCs in the presence of NBQX (10 μM) and AP-5 (50 μM) before (top trace) and after (bottom trace) JWH133 (10 μmol/L) exposure using the whole cell patch-clamp recording in SNc slice. Cumulative probability analysis for mIPSC inter-event interval and amplitude under control conditions (baseline) and JWH133 exposure. **(F–H)**. Comparison of mIPSC frequency, amplitude and half decay time before and after JWH133 exposure from SN slices tested (Amplitude: t = 0.1849, df = 9, *P* > 0.05; Frequency: t = 0.8929, df = 9, *P* > 0.05; Half decay time: t = 0.2911, df = 9, *P* > 0.05).

Based on our previous studies, we identified SNc dopaminergic neurons based on their morphological and electrophysiological characteristics: (1) SNc dopaminergic neurons are mainly located at the ventral aspect of the midbrain (Figure 1B); (2) they are densely packed, with large neuronal cell bodies, and are generally spindle, triangular, or multipole shaped (Figure 1C); (3) when dopamine neurons are stimulated with a hyperpolarized current of −150 pA (after whole-cell recordings in the current-clamp mode), significant inward rectification is observed in the neuronal membrane potential (Figure 1D); (4) In Gap free mode, SNc dopaminergic neurons exhibit slow and regular spontaneous firing activity (Figure 1E); and (5) the duration of the AP is relatively long, with a half width of about 3 ms (Figure 1F). Based on the abovementioned morphological and electrophysiological characteristics, neurons identified as dopaminergic were used in subsequent experiments.

### 3.3 Activation of CB2R on SNc dopamine neurons and inhibition of neuronal spontaneous discharge

As mentioned above, most SNc dopaminergic neurons exhibit regular tonic discharges. To observe the effect of CB2R activation on SNc dopaminergic neuronal excitability, we first selected 1 μmol/L CB2R agonist JWH133 perfusion brain slices according to previous literature reports. As demonstrated in Figure 2A, among the recorded seven dopaminergic neurons, the average firing frequency was 1.08 ± 0.13 Hz. After perfusion with JWH133, the firing frequency decreased to 0.66 ± 0.09 Hz (*P* < 0.05). The original frequency could be restored following washing with normal ACSF.

To further confirm whether the inhibitory effect of JWH133 on dopaminergic neuronal excitability was dose-dependent, brain slices perfused at four different concentrations (0.01, 0.1, 10, and 30 μmol/L) were selected. As displayed in Figure 2B: 0.01 μmol/L JWH133 demonstrated no significant effect on neuronal discharge frequency (*P* > 0.05) in the recorded display among the five neurons. In the recorded seven neurons: 0.1 μmol/L JWH133 had no obvious effect on neuron firing frequency (*P* > 0.05). However, 10 μmol/L JWH133 significantly reduced the neuron firing frequency, from the basic 1.18 ± 0.07 Hz to 0.80 ± 0.69 Hz (*P* < 0.01). Similarly, 30 μmol/LJWH133 significantly reduced neuron firing frequency, from the basic 1.11 ± 0.13 Hz to 0.69 ± 0.09 Hz (*P* < 0.01). These results indicate that the effect of JWH133 on the firing frequency of dopaminergic neurons has a significant dose correlation. Figure 2C illustrates that perfusing brain slices with 1 μmol/L of the CB2R blocker AM630 alone had no statistically significant effect on the firing frequency of dopaminergic neurons in the SNc region compared to the control group. When brain slices were perfused with both JWH133 (1 μmol/L) and AM630 (1 μmol/L), the inhibitory effect of JWH133 was blocked.

### 3.4 Activate CB2R on SNc dopamine neurons and inhibit neuronal evoked discharge

In the current-clamp mode, we administered a depolarization current stimulation of 10–50 pA to dopaminergic neurons and recorded the corresponding indicators for evoked discharges. The results demonstrated that among the seven recorded neurons, the number of AP bursts were positively correlated with increasing depolarization current amplitude (Figure 3A). In contrast, the number of APs induced by the depolarization current decreased significantly after perfusion with 10 μmol/L JWH133 (Figure 3A). To explore whether activating CB2R in the SNc brain region affects the excitability of neurons, we prepared acute SNc slices. We found that activating CB2R on dopaminergic neurons in the SNc significantly reduced neuronal excitability. Specifically, depolarizing current (50 pA, duration 800 ms) triggered larger inter-spike interval (ISI) (Figure 3B), larger afterhyperpolarization potentials (AHP), longer action potential initiation times, and fewer action potential numbers compared to the control group, indicating reduced excitability (Figures 3C–F; paired t-test, ISI: t = 4.055, df = 6, *P* < 0.01; AP initiation: t = 3.392, df = 6, *P* < 0.05; AHP: t = 3.187, df = 6, *P* < 0.05; AP duration: t = 2.403, df = 6, *P* = 0.0531; AP number: t = 5.303, df = 6, *P* < 0.01). Therefore, we concluded that activating CB2R in the SNc region reduces the intrinsic excitability of SNc dopaminergic neurons.

### 3.5 The regulation of JWH133 on miniature excitatory/inhibitory postsynaptic current

As CB2R is also expressed at pre-synapses in the SNc area, we also investigated whether JWH133 affects synaptic currents. We first examined the regulatory effects of JWH133 on mEPSCs. After whole-cell recording on mouse SNc brain slices was conducted, a set of spontaneous inward currents was obtained in the Gap free recording mode. After incorporating TTX to block the sEPSCs caused by the AP, mEPSCs were obtained. This inward current was completely blocked by NBQX and AP-5, confirming that the EPSCs were mediated by excitatory amino acid receptors (Figure 4A). As shown in Figure 4C, compared to the control group, the frequency of mEPSCs slightly decreased after the application of 10 μmol/L JWH133 (n = 10, t = 3.146, df = 9, *P* < 0.05). The half decay time of mEPSC slightly increased (n = 10, t = 2.370, df = 9, *P* < 0.05, Figure 4D). However, there was no significant difference in amplitude between mEPSC and the control group (n = 10, t = 0.09425, df = 9, *P* > 0.05, Figure 4B). This result suggests that the activation of CB2R in the SNc region alters the frequency of mEPSCs, which may be related to changes in the probability of pre-synaptic release and/or the number of synapses. We also observed an effect of JWH133 on GABA_A_ receptor-mediated mIPSCs. After the addition of NBQX (10 μmol/L) and AP-5 (50 μmol/L) to the perfused ACSF to block EPSCs caused by excitatory amino acids, IPSCs were isolated. After adding TTX (1 μmol/L) to block spontaneous IPSCs (sIPSCs) induced by APs, mIPSCs were obtained (Figure 4E). In 10 SNc slices prepared from 10 wild-type mice, 10 μM JWH133 bath perfusion for 10 min did not change the frequency, amplitude, or half decay time of mIPSCs (Amplitude: t = 0.1849, df = 9, *P* > 0.05; Frequency: t = 0.8929, df = 9, *P* > 0.05; Half decay time: t = 0.2911, df = 9, *P* > 0.05) These results suggest that the activation of CB2Rs has l effect on presynaptic GABA release or postsynaptic GABA_A_ receptor function. (Figures 4F–H).

## 4 Discussion

The dopaminergic system is involved in the regulation of many neural functions, such as motor control, spatial memory, motivation, arousal, sleep regulation, and cognitive function (Koob and Le Moal, 2008; Barishpolets et al., 2009; Keiflin and Janak, 2015). Moreover, the cannabinoid system is closely related to the dopaminergic system (Gardner, 2005; Mlost et al., 2019). CB2R is a part of the endogenous cannabinoid systems, and it has no mental side effects mediated by CB1R. Therefore, studying the role of CB2R in the dopaminergic system is necessary. CB2R is expressed in dopaminergic neurons of VTA and regulates DA release and cocaine self-administration in rats (Zhang et al., 2014). However, the effect of SNc on dopaminergic neurons has not been reported. In this study, we investigated the effects of CB2R activation on the excitability and biological function of dopaminergic neurons in substantia nigra of mice *in vitro* and *in vivo*. There were three main results. First, CB2R was expressed in SNc dopaminergic neurons of mice. Second, activation of CB2R on SNc dopaminergic neurons in mice inhibited the discharge of SNc dopaminergic neurons. This inhibitory effect existed in spontaneous discharge and when neurons were evoked by an inward current of 10–50 pA. Third, analysis of the mechanism found that this may be related to the small excitatory postsynaptic current.

Although CB2R exists in the brain, whether functional CB2R is expressed in SNc dopaminergic neurons is still unclear. Therefore, we used immunofluorescence to confirm CB2R expression in SNc dopaminergic neurons. CB2R is considered a “peripheral” receptor. However, recent studies have shown that CB2R is also widely expressed in neurons of the central nervous system (Navarro et al., 2016). Although the expression of CB2R is widespread, compared with CB1R, its function in dopaminergic neurons is less studied. In some neurons, including anteromedial medullary neurons, cultured hippocampal neurons and dopaminergic neurons of VTA, CB2R is presynaptic and inhibits the release of neurotransmitters, which seems to be complementary to the role of presynaptic CB1R (Atwood et al., 2012). Therefore, we used electrophysiological methods to explore the function of CB2R expressed on SNc dopaminergic neurons.

The whole cell patch clamp technique was used to evaluate the effect of activation of CB2R on the excitability of SNc dopaminergic neurons. JWH133 significantly inhibited the discharge of SNc dopaminergic neurons in a concentration dependent manner. This inhibitory effect was reversed by AM630, a pharmacological blocker of CB2R, indicating the presence of CB2R mediated effect. This finding is consistent with previous reports, wherein JWH133 or other CB2R agonists inhibited spontaneous and evoked neuronal discharges of VTA and inhibited excitatory neuronal discharges of the prefrontal cortex (den Boon et al., 2014; Zhang et al., 2017). Therefore, our electrophysiological data provide direct evidence that CB2R expressed in SNc dopaminergic neurons is functional, and that the activation of these receptors inhibits the discharge of SNc dopaminergic neurons and reduces the excitability of SNc dopaminergic neurons.

Action potential is the main signal transduction mechanism to activate synaptic transmission at the end of axons. The number or shape of the action potential determines the amount of calcium entering at the axon-end and the effect of synaptic transmission (Kavalali, 2015). In order to further investigate the effect of JWH133 on the inhibitory synaptic transmission of neurons, the changes of mIPSCs mediated by GABA_A_ receptor were recorded using the patch clamp technique. JWH133, a CB2R agonist, plays an important role in synaptic transmission (Kim and Li, 2015). In hippocampal CA3 pyramidal, cortical pyramidal, and VTA dopaminergic neurons, CB2 receptors reduce cell excitability through a variety of postsynaptic mechanisms, including regulation of sodium bicarbonate cotransporters (Stempel et al., 2016), and activation of chloride currents (den Boon et al., 2014) and potassium currents (Ma et al., 2019). In addition, chronic activation of CB2 receptor increased excitatory synaptic transmission and spinal density in the hippocampus through an ERK dependent mechanism (Adhikary et al., 2012). In this experiment, we observed that JWH133 could significantly reduce the frequency of mEPSCs, but had no effect on the amplitude. Moreover, JWH133 had no effect on the frequency and amplitude of mIPSCs. Thus, JWH133 inhibits the release of presynaptic glutamate to some extent and indirectly stimulates postsynaptic neurons.

In conclusion, our experimental results show that CB2R is expressed in mouse SNc dopaminergic neurons, and activation of CB2R in SNc dopaminergic neurons inhibits their excitability mainly through synaptic current mechanism. Here, we mainly discussed the regulation and possible mechanism of activation of CB2R in SNc dopaminergic neurons on neuronal excitability. These results provide a novel experimental idea for understanding the role of CB2R in the midbrain DA system and the regulation of related diseases caused by dopamine.

## Data Availability

The original contributions presented in the study are included in the article/supplementary material, further inquiries can be directed to the corresponding author.
